# The survival rate of laryngeal squamous cell carcinoma: impact of *IL1RAP* rs4624606, *IL1RL1* rs1041973, *IL-6* rs1800795*, BLK* rs13277113, and *TIMP3* rs9621532 single nucleotide polymorphisms

**DOI:** 10.1007/s12672-023-00619-0

**Published:** 2023-01-22

**Authors:** Agne Pasvenskaite, Rasa Liutkeviciene, Greta Gedvilaite, Alvita Vilkeviciute, Vykintas Liutkevicius, Virgilijus Uloza

**Affiliations:** 1 Department of Otorhinolaryngology, Lithuanian University of Health Sciences (LUHS), A. Mickeviciaus 9, LT 44307 Kaunas, Lithuania; 2 Neuroscience Institute, Lithuanian University of Health Sciences (LUHS), Kaunas, Lithuania

**Keywords:** Laryngeal squamous cell carcinoma, *IL1RAP* rs4624606, *IL1RL1* rs1041973, *IL-6* rs1800795, *BLK* rs13277113, *TIMP3* rs9621532

## Abstract

**Purpose:**

Results of laryngeal squamous cell carcinoma (LSCC) treatment and the 5 year survival rate of these patients remain poor. To purify therapeutic targets, investigation of new specific and prognostic blood-based markers for LSCC development is essential.

**Methods:**

In the present study, we evaluated five single nucleotide polymorphisms (SNPs): *IL1RAP* rs4624606, *IL1RL1* rs1041973, *IL-6* rs1800795, *BLK* rs13277113, and *TIMP3* rs9621532, and determined their associations with the patients’ 5 year survival rate. Also, we performed a detailed statistical analysis of different LSCC patients’ characteristics impact on their survival rate.

**Results:**

Three hundred fifty-three LSCC patients and 538 control subjects were included in this study. The multivariable Cox regression analysis revealed a significant association between patients’ survival rate and distribution of *IL1RAP* rs4624606 variants: patients carrying AT genotype at *IL1RAP* rs4624606 had a lower risk of death (*p *= 0.044). Also, it was revealed that tumor size (T) (*p* = 0.000), tumor differentiation grade (G) (*p* = 0.015), and *IL1RAP* rs4624606 genotype (*p* = 0.044) were effective variables in multivariable Cox regression analysis prognosing survival of LSCC patients. The specific-LSCC 5 year survival rate was 77%.

**Conclusions:**

In summary, our findings indicate that the genotypic distribution of *IL1RAP* rs4624606 influences the 5 year survival rate of LSCC patients. The results of the present study facilitate a more complete understanding of LSCC at the biological level, thus providing the base for the identification of new specific and prognostic blood-based markers for LSCC development.

## Introduction

Laryngeal Squamous Cell Carcinoma (LSCC) is a malignancy in the respiratory system, marked by the highest rate of mortality and morbidity in Head & Neck Squamous Cell Carcinoma (HNSCC) [1]. Moreover, LSCC is one of a few oncologic diseases in which the 5 year survival rate has decreased over the past 40 years, although the overall incidence is declining [2].

Three main factors could predict the outcome of LSCC: (1) host (age, sex, comorbidities, immunological response, etc.), (2) tumor (tumor site, TNM classification, differentiation grade), and obviously, (3) treatment (all available options) (3). From all these factors treatment seems to be the only controllable factor. Although there have been improvements in treatment over the last 20 years, such as endoscopic, robotic, and laser technology, however, the survival outcome, especially in advanced stages, remains unsatisfactory: the 5 year survival rate for localized LSCC is 77.4%, with spreading to regional lymph nodes–44.7%, and is crucial for outspread disease–33% [3]. According to Global Cancer Observatory data, in 2020, 99 840 cases of death caused by LSCC were registered and it is additionally about 5000 patients more than in 2018 (94 771) [4].

Not improving HNSCC patients’ survival rate might be also explained due to the absence of population-based screening programs. These programs are mostly missing because of specific examination performed by an otorhinolaryngologist–endoscopic examination of the nasopharynx, hypopharynx, and larynx. In addition, the Covid-19 pandemic had a significant impact on the delay of LSCC diagnostics [5]. Due to the lack of medical and healthcare providers, the tendency of LSCC diagnosis was observed in younger patients with larger tumor sizes [6]. For this reason, an effective appointment system with telemedicine and education programs regarding HNSCC might be useful to detect these patients earlier [7].

To improve LSCC patients’ survival recently a huge interest has been focused on the molecular landscape of LSCC willing to diagnose this disease in earlier stages and personalize the treatment [8]. For this reason, the present research analyses the genetic susceptibility of different single nucleotide polymorphisms on the survival rate of LSCC patients.

The interleukin 1 receptor accessory protein (IL1RAP), also known in the literature as IL1R3, is a co-receptor of IL-1 and IL-33 [9]. Although, the role of ILIRAP has been mainly analyzed in the context of inflammatory reactions it has been reported that IL1RAP is consistently overexpressed across multiple genetic subtypes of acute myeloid leukemia and could be a novel therapeutic target [9, 10]. However, the mechanism of *IL1RAP* rs4624606 in carcinogenesis and its impact on survival is not completely understood.

Interleukin 1 receptor-like 1(IL1RL1), also known as ST2 belongs to the interleukin 1 super-family and is also defined as the IL-33 receptor [11–13]. Recent studies have suggested that IL1RL1/IL-33 pathway may be involved in carcinogenesis [14]. Moreover, a genome-wide association study (INHANCE consortium), identified a significant role of *IL1RL1* rs1041973 in patients with different upper aerodigestive tract cancers [15]. However, the impact of *IL1RL1* rs1041973 variability is still not analyzed in these patients’ survival rates.

Interleukin-6 (IL-6) is a pleiotropic and angiogenic cytokine that affects angiogenesis, immunity, and oncological diseases [16]. The promoter of IL-6 contains several single nucleotide polymorphisms (SNPs) of which *IL6* rs1800795 historically known as *IL6*-174G/C is the most widely studied [17]. IL-6 promoter SNPs have been shown to correlate with serum and intratumoral IL-6 levels, as well as the susceptibility and survival of various carcinogenesis [18, 19]. However, the impact of *IL6* rs1800795 variants distribution on LSCC patients' survival is still not analyzed.

The B-cell lymphocyte kinase (BLK) gene has been mostly investigated in inflammatory processes and autoimmune diseases [20–22]. However, some data sources have suggested that BLK might have oncogenic properties as it could be expressed in hematological and non-haemato-logical malignancies such as breast, kidney, and lung cancers [23, 24]. To this moment there are no studies analyzing *BLK* rs13277113 gene polymorphism association with the survival of LSCC patients.

Tissue inhibitor of metalloproteinase 3 (TIMP3) is widely expressed in various tissues at a relatively high level [25]. Various studies suggest that TIMP3 has anticancer properties such as antiproliferative, antiangiogenic, and antimetastatic activities [26]. Moreover, TIMP3 might manifest as a tumor suppressor and its expression might be down-regulated in human cancer tissues [26]. The role of *TIMP3* SNPs has been analyzed in multiple cancers, however, its impact on survival is still under investigation [27–29].

According to the scientific literature, a significant role of *IL1RAP*, *IL1RL1*, *IL-6, BLK*, and *TIMP3* in carcinogenesis has been already suggested [9, 10, 14, 15, 18, 19, 23, 24, 27–29]. However, to the best of our knowledge, no comprehensive studies analyzing the impact of these genes’ SNPs distribution on LSCC patients’ survival have been published so far. For this reason, we have selected the most analyzed in scientific literature SNPs of each gene-*IL1RAP* rs4624606, *IL1RL1* rs1041973, *IL-6* rs1800795, *BLK* rs13277113, and *TIMP3* rs9621532, to investigate their associations with LSCC patients’ 5 year survival rate. We believe that our findings could contribute to a better understanding of LSCC pathogenesis at the molecular level and the selection of blood-based molecular marker complexes required for the diagnosis and prognosis of LSCC.

## Materials and methods

The present case-control study was conducted at the Department of Otorhinolaryngology, Lithuanian University of Health Sciences (LSMU), Kaunas, Lithuania, and at the Laboratory of Ophthalmology, Neuroscience Institute, LSMU, Kaunas, Lithuania, 2009–2021.

### Ethics statement

The study protocol was confirmed by Kaunas Regional Ethics Committee for Bio-medical Research, LSMU (authorization number BE-2-37). All studies concerning procedures were accomplished following the Declaration of Helsinki. All participants were informed about the structure and objectives of the present study before its launch. An Informed Consent Form was obtained from all subjects involved in the study.

### Study population

891 subjects were enrolled in this study: 353 patients with LSCC, and 538 healthy controls as a reference group. Characteristics of study groups are presented in Table 1. Data on age, sex, smoking habits, and alcohol consumption were compared between the LSCC and control groups. The control group was adjusted by sex and age to the LSCC group (p = 0.985; p = 0.091, respectively).

**Table 1:** Demographic characteristics of the study

Characteristic	Group		*p-*Value^3^
	LSCC^1^ n = 353	Control group n = 538	
Male, n (%)	338 (95.8)	515 (95.7)	0.985^a^
Female, n (%)	15 (4.2)	23 (4.3)	
Age years; median (IQR)^2^	63 (10)	63 (10)	0.091^b^
Smoking, n			< 0.001
Yes	132 (37.4)	36 (6.7)	
No	5 (1.4)	166 (30.9)	
Unknown	216 (61.2)	336 (62.4)	
Alcohol consumption^c^, n			< 0.001
Yes	107 (30.3)	108 (20.1)	
No	30 (8.5)	94 (17.5)	
Unknown	216 (61.2)	336 (62.4)	
Stage, n (%)			–
I	115 (32.6)		
II	76 (21.5)		
III	61 (17.3)		
IV	101 (28.6)		

^1^*LSCC* Laryngeal Squamous Cell Carcinoma, ^2^*IQR* interquartile range, ^3^*p-Value* significance level *p* < 0.05

^a^Pearson Chi-Square

^b^Student’s *t*-test

^c^Data about smoking and alcohol consumption were collected from 137 LSCC patients and 202 control group subjects

### Selection of study population

The study population was composed of two groups: the LSCC group and the healthy controls.

#### LSCC group

A comprehensive otorhinolaryngological examination including flexible endoscopy and/or video laryngostroboscopy and was carried out for all patients with suspected LSCC at the Outpatient Office of the Department of Otorhinolaryngology, LSMU. Collection of peripheral venous blood samples was performed from the catheter inserted to induce general anesthesia. All the patients underwent direct micro laryngoscopy with biopsy. The histological diagnosis of LSCC was confirmed at the Department of Pathology, LSMU. Other important clinical data were obtained through reviews of patients’ case records and personal interviews. Data about harmful habits were collected from the unified questionnaire used in the Health Behavior Among Lithuanian Adult Population, 2012 project (questions from numbers 50 to 62 were selected for the study) [30]. To determine the final diagnosis with staging, laryngeal, and neck CT scans or/and MRI were performed. The staging of LSCC was done following the Guidelines for Head and Neck Cancers Classification, Version 2.2020 accepted by National Comprehensive Cancer Network (NCCN) [31].

Patients diagnosed with another type and localization of cancer, acute or chronic infectious diseases, individuals using psychomotor suppressants and antiepileptic drugs, pregnant or breastfeeding women, and persons younger than 18 years old (according to Convention on the Rights of the Child (Lithuania acceded in 1992), every human being below the age of 18 means a child if his or her adulthood has not been recognized before by law) were excluded from this study.

#### Healthy controls

Patients who presented at the otorhinolaryngologist’s consultation at the Out-patient Office at the Department of Otorhinolaryngology, LSMU, and were selected for surgical treatment (tympanoplasty, ossiculoplasty, tympanostomy, nasal bones reposition, septoplasty, rhinoseptoplasty, uvulopalatopharyngoplasty, or radiofrequency thermoablation) were enrolled into the present study. Peripheral venous blood samples were collected from the same catheter inserted to induce general anesthesia. Also, patients who presented at the family doctor's consultation for a general check-up and had a complete blood count test were enrolled in this study.

Patients with the diagnosed oncologic disease, acute or chronic infectious diseases, individuals using psychomotor suppressants and anti-epileptic drugs, pregnant or breastfeeding women, and persons younger than 18 years old were excluded from this study.

### Deoxyribonucleic acid extraction

Deoxyribonucleic acid (DNA) extraction was carried out at the Laboratory of Ophthalmology, Neuroscience Institute, LSMU. Peripheral venous blood samples were collected in 200 μL EDTA-containing vacutainer tubes and stored at − 80 °C until the DNA extraction procedure.

The genomic DNA was extracted using silica-based membrane technology, using a genomic DNA extraction kit (GeneJET Genomic DNA Purification Kit, Thermo Fisher Scientific, Vilnius, Lithuania), based on the manufacturer’s recommendations.

### Genotyping

The analysis of* IL1RAP* rs4624606,* IL1RL1* rs1041973, *IL-6* rs1800795, *BLK* rs13277113, and *TIMP3* rs9621532 was carried out at the Laboratory of Ophthalmology, Neuroscience Institute, LSMU. The genotyping of *IL1RAP* rs4624606, *IL1RL1* rs1041973, *IL-6* rs1800795, *BLK* rs13277113, and *TIMP3* rs9621532 was performed using the real-time polymerase chain reaction (PCR) method. Identification of all single-nucleotide polymorphisms was performed using TaqMan^®^ Genotyping assays (Thermo Fisher Scientific, Inc, Pleasanton, USA). The genotyping was performed using a “StepOnePlus” real-time PCR quantification system (Thermo Fisher Scientific, Singapore). Results on individual genotypes were obtained using the Allelic Discrimination program.

### Quality control of genotyping

The repetitive analysis of 5% randomly chosen samples was performed for all five SNPs to confirm the same rate of genotypes from initial and repetitive genotyping.

### Survival rate

The LSCC group data about the mortality rate, including the survival period after diagnosis of LSCC, and the cause of death was collected from the Lithuanian State Register of Death Cases and Their Causes.

### Statistical analysis

Data on demographic characteristics of study participants were compared between control group subjects and the LSCC group using the Pearson Chi-square test and Student’s t-test and presented as absolute numbers with percentages in brackets. The frequencies of all selected SNPs (*IL1RAP* rs4624606, *IL1RL1* rs1041973, *IL-6* rs1800795, *BLK* rs13277113, and *TIMP3* rs9621532) genotypes and alleles are presented in percentages.

To compare the observed and expected frequencies of selected SNPs in the control group, the analysis of Hardy–Weinberg using the Chi-square test was performed. The Chi-square test was used to compare the distribution of *IL1RAP* rs4624606, *IL1RL1* rs1041973, *IL-6* rs1800795, *BLK* rs13277113, and *TIMP3* rs9621532 in the LSCC and control groups.

Binomial logistic regression analysis with an adjusted odds ratio (OR) and its 95% confidence interval (95% CI) was utilized to evaluate the influence of selected SNPs genotypes and alleles on LSCC development, and the risk prediction for LSCC patients with these polymorphisms. The binomial logistic regression analysis results are represented as genetic models: codominant, dominant, recessive, overdominant, and additive. The best genetic model selection was based on the Akaike Information Criterion (AIC); therefore, the best genetic models were those with the lowest AIC values.

LSCC patients’ survival analysis was accomplished using the Life-Table method. To compare survival rates in different subgroups, Gehan’s criterion was utilized. To determine the impact of different variables on the risk of dying from LSCC, the univariate and multivariate Cox regression proportional hazard models were used. Different variables were evaluated by analyzing them one by one and as a whole of variables. Hazard ratios and their 95% confidence intervals were calculated.

Statistical analysis was performed using the SPSS/W 22.0 software (Statistical Package for the Social Sciences for Windows, Inc., Chicago, IL, USA). The findings were considered statistically significant when p < 0.05.

## Results

### SNP analysis

After performing statistical analysis, it was revealed significant associations between *TIMP3* rs96215332 variants and LSCC in the codominant (OR = 0.600; 95% CI 0.390–0.922;* p* = 0.020), overdominant (OR = 0.599; 95% CI 0.390–0.922; *p* = 0.020) and additive (OR = 0.675; 95% CI 0.459–0.991; *p* = 0.045) models. Also, significant associations between *IL1RAP* rs4624606 variants and LSCC were determined in the codominant (OR = 1.372; 95% CI 1.031–1.827; *p* = 0.030), overdominant (OR = 1.353; 95% CI 1.018–1.798; *p* = 0.037) and additive (OR = 1.337; 95% CI 1.038–1.724; *p* = 0.025) models. More detailed information on this matter is available elsewhere [32].

### Survival analysis

The 5 year overall survival (OS) rate of selected 353 LSCC patients including all causes of death was 67%. The percentage of the LSCC group who survived precisely from LSCC diagnosis date–LSCC-specific survival–to a period of 5 year was 77% (patients who died from causes unrelated to LSCC were not included in this measurement).

Different characteristics of LSCC patients were analyzed one by one, without excluding the influence of other variables. Table 2 presents the impact on participants’ characteristics and 1-, 3- and 5 year survival rates among LSCC patients.

**Table 2:** Characteristics of LSCC patients and impact on survival rate

Variable		N = 353 (%)	Survival rate (%)			*p*-value^2a^
			1 Year LSCC^1^-specific survival (%)	3 Year LSCC^1^-specific survival (%)	5-Year LSCC^1^-specific survival (%)	
Age	< 60 year-old	119 (33.7)	87	76	74	0.705
	60–69 year-old	151 (42.8)	87	81	76	
	≥ 70 year-old	83 (23.5)	84	73	73	
T^3^	1	132 (37.4)	96	94	94	0.000
	2	68 (19.3)	86	67	64	
	3	70 (19.8)	82	71	63	
	4	83 (23.5)	75	66	63	
N^4^	0	85 (24.1)	91	83	80	0.000
	≥ 1	268 (75.9)	69	57	53	
G_5_	1	107 (30.3)	89	80	77	0.022, 0.008^b^, 0.013^c^
	2	200 (56.7)	88	80	76	
	3	46 (13.0)	69	57	57	
Stage	I	122 (34.6)	96	94	94	0.000
	II	73 (20.7)	85	68	65	
	III	63 (17.8)	83	71	64	
	IV	95 (26.9)	76	67	64	
Smoking^d^	< 25 years	38 (27.5)	89	87	76	0.034
	≥ 25 years	100 (72.5)	84	63	58	
Alcohol consumption^d^	Yes	107 (77.5)	90	73	73	0.270
	No	31(22.5)	84	69	61	
Treatment option^e^	Surgical	188 (71.8)	88	82	80	0.008
	Nonsurgical (RT^6^, CRT^7^)	74 (28.2)	76	67	66	
Type of surgery	Laryngectomy with/without ND^8^	67 (35.6)	68	66	62	0.000
	Partial laryngectomy with/without ND	45 (24.0)	91	86	81	
	Cordectomy with/without ND	76 (40.4)	100	94	94	
Primary/Recurrence	Primary	224 (85.5)	86	79	78	0.400
	Recurrence	38 (14.5)	81	71	65	

^1^*LSCC* laryngeal squamous cell carcinoma, ^2^*p-Value* significance level *p* < 0.05, ^3^*T* tumor size, ^4^*N* metastasis to lymph nodes, ^5^*G* tumor differentiation grade, ^6^*RT* radiotherapy, ^7^*CHT* chemoradiotherapy, ^8^*ND* neck dissection

^a^Gehan’s criterion

^b^G3 *vs.* G1

^c^G3 *vs.* G2

^d^Data about smoking and alcohol consumption were collected from 137 LSCC patients

^e^Data about treatment option and primary/recurrence cases were collected from 262 LSCC patients

Patients’ survival rate correlated statistically significantly with T (tumor size according to the TNM classification): the 5 year survival rate for patients with T1 (94%) was statistically significantly improved compared with T2 (64%), T3 (63%), or T4 (63%), (*p* = 0.000). Patients with spreading of the disease to the neck lymph nodes (N ≥ 1) had a statistically significantly poorer 5 year survival rate (53%), compared to those who did not have metastasis to the neck lymph nodes (80%) (*p* = 0.000). Moreover, patients with a higher differentiation grade of the tumor (G) had a lower 5 year survival rate (*p* = 0.022): G3 survival rate was lower compared to patients with G1 and G2 (*p* = 0.008 and *p* = 0.013, respectively). In addition, the 5 year survival rate of patients with stage I (94%) was statistically significantly higher compared to II, III, and IV stages (65%, 64%, and 64%, respectively), (*p* = 0.000).

The 5 year survival rate of patients who had a longer (≥ 25 years) smoking experience was lower (58%) compared to those who had a shorter (< 25 years) smoking experience (76%), (*p* = 0.034). However, we didn’t find any statistically important differences in survival rate between patients who consumed alcohol and those who did not (*p* = 0.270).

We identified that patients who underwent surgical treatment had a better 5 year survival rate (80%) compared to those who were treated with radiotherapy or chemoradiotherapy (66%) (*p* = 0.008). Furthermore, we clarified that surgical treatment was used more often for patients who did not have metastasis to the neck lymph nodes *(p* = 0.003). In cases with neck metastasis, total laryngectomy was performed more often (*p* = 0.000).

Also, in our selected LSCC group patients with well-differentiated tumors (G1–82.2%) were submitted to surgical treatment more often compared to intermediate grade (G2–67.9%) and poor differentiation grade (G3–66.7%), (*p* = 0.008). Patients with well-differentiated tumors (G1 or G2) underwent more often cordectomy, and patients with G3 were submitted more often to total laryngectomy (*p* = 0.002). In the case of LSCC recurrence, laryngectomy was performed more often compared to partial laryngectomy (*p* = 0.003).

Table 3 represents the impact on survival rate according to the genotypic distribution of selected SNPs.

**Table3:** The genotypic distribution of *IL1RAP* rs4624606, *IL1RL1* rs1041973, *IL-6* rs1800795, *BLK* rs13277113, and *TIMP3* rs9621532 SNPs according to the 1-, 3- 5 year survival rate

Polymorphism	Genotype	N = 353 (%)	Survival rate (%)			*p*-value^2a^
			1 Year LSCC^1^-specific survival (%)	3 Year LSCC^1^-specific survival (%)	5 Year LSCC^1^-specific survival (%)	
*IL1RAP* rs4624606	AA	26 (7.4)	89	77	39	0.107 0.038^b^
	AT	126 (35.7)	90	78	70	
	TT	201 (56.9)	80	65	63	
*IL1RL1* rs1041973	AA	37 (10.5)	85	70	52	0.854
	AC	132 (37.4)	81	69	62	
	CC	184 (52.1)	86	70	69	
*IL-6* rs1800795	CC	109 (30.9)	86	74	72	0.511
	CG	162 (45.9)	84	69	61	
	GG	82 (23.2)	80	66	63	
*BLK* rs13277113	AA	31 (8.8)	79	64	64	0.802
	AG	140 (39.7)	84	72	69	
	GG	182 (51.5)	84	69	62	
*TIMP3* rs9621532	AA	290 (82.2)	83	70	64	0.490
	AC	43 (12.2)	92	68	68	
	CC	20 (5.6)	1	1	1	

^1^*LSCC* Laryngeal squamous cell carcinoma; ^2^*p-Value* significance level *p* < 0.05

^a^Gehan’s criterion

^b^AT *vs.* AA and TT

**Fig. 1:**
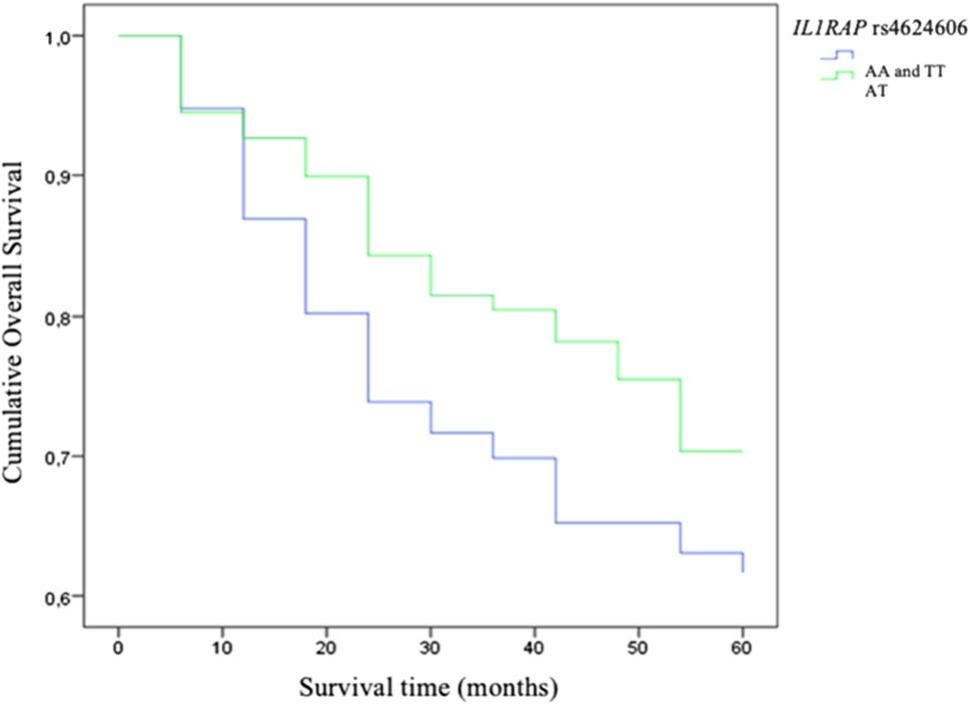
The 5 year survival rate according to the distribution of *IL1RAP* rs4624606 genotypes

Analyzing LSCC patients’ 5 year survival rate and the genotype distribution, we clarified that subjects carrying AT genotype at *IL1RAP* rs4624606 had a statistically significantly better 5 year survival rate (70%) than those who carry AA and TT genotypes (39% and 63%, respectively), (*p* = 0.038), (Fig. 1).

The univariate Cox proportional Hazard model (Table 4), identified that tumor size (T), metastasis to the neck lymph nodes (N), tumor differentiation grade (G), stage, and treatment option was effective variables in the survival of LSCC patients. It was clarified that each year of patients’ age increases the risk of mortality 1.012 times, however, not statistically significantly (*p* = 0.766). As the result, patients with T2, T3, and T4 had 6.575, 6.678, and 7.666 times increased risk of death respectively, compared to patients with T1 (HR_T2 *vs.* T1_ = 6.575, 95% CI 2.647–17.268, *p* = 0.000; HR_T3 *vs.* T1_ = 6.678, 95% CI 2.652–16.781, *p* = 0.000; HR_T4 *vs.* T1_ = 7.666, 95% CI 3.214–18.677, *p* = 0.000). The risk of death in patients who had metastasis to the neck lymph nodes (N ≥ 1) was 2.998 times higher than those without metastasis to the neck lymph nodes (N = 0) (HR_N≥1 *vs.* N=0_ = 2.998, 95% CI 1.823–4.847, *p* = 0.000). According to tumor differentiation grade (G), it was clarified that patients with G3 have a statistically significantly higher risk of death compared to patients with G1 and G2 (HR_G3 *vs.* G1_ = 2.618, 95% CI 0.141–0.842, *p* = 0.021; HR_G3 *vs.* G2_ = 2.433, 95% CI 0.243–0.828, *p* = 0.010). According to the stage, the risk of death in patients with II, III, and IV stages was higher than in those with stage I tumors (HR_II *vs.* I_ = 6.738, 95% CI 2.723–16.654, *p* = 0.000; HR_III *vs.* I_ = 6.459, 95% CI 2.591–16.569, *p* = 0.000; HR_IV *vs.* I_ = 7.172, 95% CI 2.858–17.361, *p* = 0.000). Patients who had ≥ 25 years of smoking experience had 2.372 times increased risk of death compared to those who were smoking for less than 25 years (HR_25≥years *vs*. <25 years_ = 22.372, 95% CI 0.915–6.147, *p* = 0.046). However, alcohol consumption increased the risk of death statistically insignificantly (HR_Users *vs.* abstinent_ = 1.670, 95% CI 0.644–4.328, *p* = 0.274). Besides, patients who underwent surgical treatment faced a 1.949 times lower risk of death compared to those who received nonsurgical treatment (HR_Surgical *vs.* Nonsurgical_ = 0.513, 95% CI 0.302–0.871, *p* = 0.013). The patients who had a recurrence of the disease had a statistically insignificantly higher risk of death compared to those who had LSCC diagnosed for the first time (HR_Relapse *vs.* Primary_ = 1.474, 95% CI 0.763–2.846, *p* = 0.248).

**Table 4:** Association between study variables and LSCC patients’ mortality in univariate *Cox* proportional Hazard model

Variable		HR^1^	Univariate 95% CI^2^	*p*-value^6^
Age		1.012	0.976–1.036	0.766
T^3^	T2 *vs.* T1	6.575	2.647–17.268	0.000
	T3 *vs.* T1	6.678	2.652–16.781	0.000
	T4 *vs.* T1	7.666	3.214–18.677	0.000
N^4^	N ≥ 1 *vs.* N = 0	2.998	1.823–4.847	0.000
G^5^	G3 *vs*. G1	0.382	0.141–0.842	0.022
	G2 *vs*. G2	0.411	0.243–0.828	0.010
Stage	II *vs.* I	6.738	2.723–16.654	0.000
	III *vs.* I	6.459	2.591–16.569	0.000
	IV *vs.* I	7.172	2.858–17.361	0.000
Smoking ≥ 25 years *vs.* < 25 years		2.134	0.815–6.263	0.046
Alcohol users *vs*. Non-drinkers		1.560	0.638–4.224	0.274
Treatment surgical *vs*. non-surgical		0.513	0.302–0.871	0.013
Tumor relapse *vs.* primary		1.474	0.763–2.846	0.248
*IL1RAP* rs4624606		1.506	0.969–2.341	0.069
*IL1RL1* rs1041973		1.295	0.613–2.734	0.718
*IL-6* rs1800795		0.708	0.397–1.264	0.243
*BLK* rs13277113		1.115	0.446–2.788	0.816
*TIMP3* rs9621532		0.735	0.340–1.587	0.433

^1^*HR* hazard ratio, ^2^*CI* confidence interval, ^3^*T* tumour size, ^4^*N* metastasis to the neck lymph nodes, ^5^*G* tumour differentiation grade, ^6^*p-Value* significance level *p* < 0.05

To analyze the whole variables, a multivariable Cox proportional hazard model was utilized. Variables that were analyzed one by one previously and had a significant *p*-Value were involved. To obtain an optimal model, the Backward Stepwise method was applied. Referring to the results after the Backward Stepwise method (the final multivariable Cox proportional hazard model), only three significant predictors remained: tumor size (T), tumor differentiation grade (G), and *IL1RAP* rs4624606 genotype (Table 5). Moreover, although in univariable analysis inspecting different variables one by one, metastasis to the neck lymph nodes (N) (N ≥ 1 *vs.* N = 0), stage (I vs. II, III, and IV), and treatment option (surgical *vs*. non-surgical) were significant for patients’ survival rate, these characteristics were not significant in the multivariate analysis.

**Table 5:** Association between study variables and LSCC patients’ mortality in the final multivariable *Cox* proportional hazard model after the Backward Stepwise method

Variable		HR^1^	Multivariable 95% CI^2^	*p*-value^5^
T^3^	T2 *vs.* T1	2.937	1.625–5.644	**0.000**
	T3 *vs.* T1	3.016	1.684–5.745	**0.000**
	T4 *vs.* T1	3.038	1.705–5.676	**0.000**
N^4^	G3 *vs.* G1and G2	2.062	1.247–3.878	**0.015**
*IL1RAP* rs4624606 AT *vs.* AA and TT		1.590	1.013–2.495	**0.044**

^1^*HR* hazard ratio, ^2^*CI* confidence interval, ^3^*T* tumour size, ^4^*N* metastasis to the neck lymph nodes, ^5^*p-Value* significance level *p* < 0.05

In multivariable analysis, it was identified, that patients with T2, T3, and T4 had an increased risk of death, compared to patients with T1 (HR_T2 *vs.* T1_ = 2.937, 95% CI 1.625–5.644, *p* = 0.000; HR_T3 *vs.* T1_ = 3.016, 95% CI 1.684–5.745, *p* = 0.000; HR_T4 *vs*. T1_ = 3.038, 95% CI 1.705–5.676, *p* = 0.000). Moreover, the risk of death in patients who had tumor differentiation grade G3 was higher than in those with G1 or G2 (N = 0) (HR_G3 *vs.* G1_ and G2 = 2.062, 95% CI 1.247–3.878, *p* = 0.015). Furthermore, it was clarified that patients carrying AT genotype at *IL1RAP* rs4624606 have a 1.590 times lower risk of death compared to those who carry AA and TT genotypes (HR_AT *vs*. AA_ and TT = 1.590, 95% CI 1.013–2.495, *p* = 0.044).

## Discussion

In the present study, we investigated if the genetic distributions of *IL1RAP* rs4624606, *IL1RL1* rs1041973, *IL-6* rs1800795, *BLK* rs13277113, and *TIMP3* rs9621532 SNPs are associated with LSCC patients’ 5 year survival rate. The results of our study identified that the genetic distribution of *IL1RAP* rs4624606 has a significant impact on the 5 year survival rate for LSCC patients: patients carrying AT genotypes at *IL1RAP* rs4624606 have a 1.590 times lower risk of death. Moreover, *IL1RAP* rs4624606 AT genotype remained in the optimal multivariate Cox proportional hazards model demonstrating a significant role in the prediction of LSCC survival (*p* = 0.044).

The present study also analyzed the impact of different LSCC characteristics and treatment options on LSCC patients’ survival rates. This study revealed that patients with T4 (*p *= 0.000), metastasis to the neck lymph nodes (*p* = 0.000), and higher tumor differentiation grade G (G3) (*p* = 0.013) had a statistically significant lower 5 year survival rate. In addition, tumor size (T), metastasis to neck lymph nodes (N), and tumor differentiation grade (G) persisted in the optimal multivariate Cox proportional hazards model. To date, all these findings correspond to data in the literature [33]. However, the biological aggressiveness of the tumor and the immunological response of the host are not associated with the TNM classification system. Moreover, TNM classification is not oriented to the selection of personal treatment and clinical evaluation of LSCC that should also include biological markers which might broaden the knowledge of tumor behavior [3].

Predicting the probable outcome for individual LSCC patients remains challenging. Moreover, meanwhile, doctors are focused on selecting an optimal treatment, and patients are much more worried about their prognosis. In this study, we analyzed the well-recognized risk factors of LSCC–smoking and alcohol consumption, believing that these factors might be significant in individual prognosis. According to the literature, tobacco use and alcohol consumption are the strongest risk factors for the development of LSCC as nearly 95% of patients with LSCC have a smoking or alcohol consumption history [34]. In the present study, we revealed that the 5 year survival rate of patients who had a longer (≥ 25 years) smoking experience was statistically significantly lower (58%) than for those who had a shorter (< 25 years) smoking experience (76%), (*p* = 0.034). Moreover, patients who have smoked longer than 25 years had a 2.134 times higher risk of dying from LSCC. However, we did not find any significant differences between alcohol consumers and non-drinkers when analyzing survival rate differences *(p* = 0.274). Interestingly, while the majority of LSCC patients can be considered as affected by harmful factors, the etiology behind the development of this particular tumor in non-smoking patients is still unrevealed [35]. The study conducted by Malm *et* al. provides clear evidence that non-smokers with LSCC tend to be younger and more likely to have early-stage glottic cancer. Furthermore, in their study, they concluded that the molecular and immunologic characteristics of non-smokers and smokers are similar [34]. In addition, Shoffel-Havahuk *et* al. in their research identified that non-smoking female patients with a bimodal age distribution (< 40 years or > 75 years at diagnosis) represent a significantly higher rate of LSCC incidence than smoking female patients [36]. This information requires attention to consider revising the traditional screening paradigm. Another study, performed by Huang *et al.* also demonstrated that non-smoking and non-alcohol-drinking female patients are more likely to represent LSCC cases and more at the earlier stage of the disease [37]. Moreover, the results of the study suggested that non-smoking and non-alcohol-drinking LSCC patients not only have the same rate of disease recurrence as smoking and alcohol-drinking patients, but they also have a comparable disease outcome. Bearing this in mind, understanding of LSCC cell composition and molecular characteristics could contribute to improving the role of risk factors on LSCC etiology and developing adequate screening programs of HNSCC patients.

Due to a lack of effective molecular targeting therapy, LSCC presents a static or even slightly decreased survival rate [38]. In addition, the correct and appropriate treatment choice for LSCC patients during multidisciplinary team meetings remains challenging as attention has to be paid to patients’ speech, swallowing, and breathing functions that impact their quality of life and psychosocial status [39]. In the present study, the impact of treatment choice on LSCC patients’ 5 year survival rate also was investigated. It was revealed that patients who underwent surgical treatment had a better 5 year survival rate than those who received radiotherapy and/or chemotherapy (overall survival rate: 80% *vs.* 66%, *p* = 0.008 respectively). Moreover, surgical treatment was applied more often to the patients who did not have metastasis to the neck lymph nodes (*p* = 0.003). In addition, total laryngectomy was performed more often in a case with metastasis to the neck lymph nodes (*p* = 0.000). These results depend on patients’ distribution by tumor stage–the early stage of LSCC (I and II stages) was predominant and confirmed in 195 LSCC patients (55.2%) who were treated surgically. However, 158 (44.8%) patients with an advanced LSCC (III and IV stages) were treated surgically or non-surgically (radiotherapy or/and chemotherapy) as was decided by the multidisciplinary team. Therefore, it is complicated to collect large cohorts of homogenous tumors (stage and site) that would be treated with the same modality [40].

To date, no tools could predict the evolution of LSCC. For this reason, many studies investigate the role played by different biomarkers expressed by laryngeal cancer. However, these biomarkers are not as well defined as in the other cancer types such as lung, colon, or breast and often have controversial results [39]. In this study, we investigated five different SNPs (*IL1RAP* rs4624606, *IL1RL1* rs1041973, *IL-6* rs1800795, *BLK* rs13277113, and *TIMP3* rs9621532). The selection of these SNPs was based on their role in carcinogenesis discovered in previous research [10, 14, 18, 19, 23, 24, 26–29]. In addition, our previous study results demonstrating a significant association between *IL1RAP* rs4624606 and *TIMP3* rs9621532 SNPs in LSCC carcinogenesis seemed to be promising [32]. However, after a detailed analysis of the impact on the survival rate of LSCC patients we identified that only *IL1RAP* rs4624606 variants’ genetic distribution seems to be a significant prognostic factor in LSCC development. However, our negative results of *IL1RL1* rs1041973, *IL-6* rs1800795, *BLK* rs13277113, and *TIMP3* rs9621532 variants’ genetic distribution impact on LSCC survival are also meaningful and important not to be repeated in further investigations. Therefore, both positive and negative results are essential to make the scientific process robust and credible.

The low survival rate of LSCC patients also could be explained by the absence of population-based screening programs for HNSCC patients. Public awareness and education are necessary for improving LSCC prevention and early malignancy detection [42]. Moreover, HNSCC screening programs need to target patients with recognizable risk factors [43]. Scheduled and opportunistic screening by appropriately trained individuals is highly recommended for targeted populations [44]. In addition, the unknown prognosis of diagnosed oncological disease causes patients’ anxiety, consequently, the suicide mortality rate among patients with head and neck cancer is high–59, 64, and 127 per 100 000 person-years among residents of metropolitan, urban, and rural counties, respectively [45]. Therefore, a brief psychosocial screening protocol should be implemented in routine ambulatory oncology care as 57% of HNSCC patients were identified with clinical distress [46]. Finally, a better understanding of LSCC cellular heterogeneity could contribute to improving earlier diagnostics and survival rates of LSCC patients [47].

The strength of this study is (1) the large study population involvement (891 subjects in total); (2) the careful selection of investigated groups (the LSCC patients and control groups were adjusted for age and sex); (3) the involvement of harmful risk factors (smoking and alcohol consumption), (4) data collection from single tertiary center, (5) enrolment of a pure LSCC cohort, (6) and the clinical significance (prognosis of LSCC patients’ survival). According to the literature, most genetic studies present data united under the umbrella of the HNSCC term, including malignant tumors of different localizations (oral, pharyngeal, nasopharyngeal, hypopharyngeal, laryngeal regions, etc.) and not taking into account that these malignancies have different etiology, biological and clinical behavior, and distinct genomic profiles [48]. Moreover, we believe that fusing different localization HNSCCs may mask possible meaningful associations of selected biomarkers with individual cancer types.

To the best of our knowledge, this is the first report that associates the role of *IL1RAP* rs4624606, *IL1RL1* rs1041973, *IL-6* rs1800795, *BLK* rs13277113, and *TIMP3* rs9621532 variants distribution with LSCC patients’ 5 year survival rate in wide-ranging, pure and homogenous LSCC patients’ cohort and age- and sex equivalent control subjects. This peculiarity permitted us to perform an accurate analysis of associations between selected SNPs and the survival rate of LSCC patients with a particular tumor in a specific head and neck anatomical region. As reported by Cadoni *et* al. LSCC behavior is presented as less aggressive compared to other HNSCC, assuming a rather low metastatic rate and local spreading [48]. Therefore, the results of the present study demonstrate the absence of differences *in IL1RL1* rs1041973, *IL-6* rs1800795, *BLK* rs13277113, and *TIMP3* rs9621532 variants distribution between LSCC patients and control subjects are comprehensible. Nevertheless, a statistically significant association of AT genotype at *IL1RAP* rs4624606, with a better LSCC-specific 5 year survival rate, suggests this SNP’s clinical significance demonstrating a tendency for better prognosis in choosing treatment modalities (surgical *vs.* non-surgical treatment).

## Conclusion

Results of the present study indicate a significant association between AT genotype at *IL1RAP* rs4624606 and LSCC development and are associated with a better 5 year survival rate. These results demonstrate clinical significance and might be useful for selecting candidate prognostic factors in identifying and elaborating novel targeted therapy strategies and could improve LSCC patients’ survival rate. However, further research is required before it can be used as a therapeutic target.

## Data Availability

The datasets generated during and/or analyzed during the current study are available from the corresponding author on reaonable request.
